# Patterns of Positive Selection and Neutral Evolution in the Protein-Coding Genes of *Tetraodon* and *Takifugu*

**DOI:** 10.1371/journal.pone.0024800

**Published:** 2011-09-13

**Authors:** Juan I. Montoya-Burgos

**Affiliations:** Department of Genetics and Evolution, University of Geneva, Geneva, Switzerland; Biodiversity Insitute of Ontario-University of Guelph, Canada

## Abstract

Recent genome-wide analyses have revealed patterns of positive selection acting on protein-coding genes in humans and mammals. To assess whether the conclusions drawn from these analyses are valid for other vertebrates and to identify mammalian specificities, I have investigated the selective pressure acting on protein-coding genes of the puffer fishes *Tetraodon* and *Takifugu.* My results indicate that the strength of purifying selection in puffer fishes is similar to previous reports for murids but stronger in hominids, which have a smaller population size. Gene ontology analyses show that more than half of the biological processes targeted by positive selection in mammals are also targeted in puffer fishes, highlighting general patterns for vertebrates. Biological processes enriched with positively selected genes that are shared between mammals and fishes include immune and defense responses, signal transduction, regulation of transcription and several of their descendent terms. Mammalian-specific processes displaying an excess of positively selected genes are related to sensory perception and neurological processes. The comparative analyses also revealed that, for both mammals and fishes, genes encoding extracellular proteins are preferentially targeted by positive selection, indicating that adaptive evolution occurs more often in the extra-cellular environment rather than inside the cell. Moreover, I present here the first genome-wide characterization of neutrally-evolving regions of protein-coding genes. This analysis revealed an unexpectedly high proportion of genes containing both positively selected motifs and neutrally-evolving regions, uncovering a strong link between neutral evolution and positive selection. I speculate that neutrally-evolving regions are a major source of novelties screened by natural selection.

## Introduction

Protein evolution is the outcome of the interplay between mutational processes and selective forces acting at the molecular level and, therefore, analyzing the coding fraction of a genome is fundamental for understanding how natural selection influences the evolution of organisms. It is generally assumed that functional elements of the genome, either coding or non-coding, show conserved sequences among related species due to purifying selection (Dermitzakis et al, 2002). During evolution, such elements have presumably reached functional local optima *via* adaptive evolution and thereafter, most mutations that modified the function must have been discarded by purifying selection. Accordingly, genes with a low rate of evolution must have central functions which they are fulfilling in an optimal (or nearly-optimal) manner and the probability of performing better is very low unless the interaction network to which they belong is modified. Purifying selection is the main driving evolutionary force currently acting on those genes. In mammals, at least 75% of non-synonymous substitutions are removed by purifying selection [1], [2]. On the contrary, genes that have less central functions tend to evolve at higher rates and a fraction of them may have experienced recent positive selection. Supporting this principle, it has been suggested that the evolutionary rate of protein-coding genes depend on protein dispensability [3] or on protein connectivity [4], [5], [6]. Accordingly, the central function of housekeeping genes may explain why they evolve at a slower rate than tissue-specific genes [7]. Moreover, positive selection tends to act more often on proteins with lower protein-protein connectivity [8]. Likewise, there are significantly more Positively Selected Genes (PSG) in the fraction of duplicated or paralogous genes with redundant information than in the fraction of single-copy genes [9]. The probability of undergoing either purifying selection or positive selection is thus closely linked to the biological function or functional redundancy of the gene product.

In various genome-wide analyses of selection acting on protein-coding genes, positive selection has been shown to impact more often genes involved in particular biological functions. The functions related to immunity, defense and sensory perception have been shown to be preferential targets of positive selection in humans and other mammals [2], [10], [11]. In humans, it has also been suggested that spermatogenesis, apoptosis-related and cancer-related gene categories are enriched with PSG [12], [13].

Although genome-wide analyses of selective pressure have provided important knowledge, shedding light on major patterns of positive selection, they are still limited by weak statistical power to discriminate between positive selection and neutral evolution. In most studies reporting genome-wide analyses of selection acting on protein-coding genes, the methodological approach was to use the criterion of Ka/Ks>1 (or dN/dS>1) over the full-length coding sequence (CDS) to infer positive selection. If the species analyzed are close relatives, this approach returns a limited number of significant results due to a reduced amount of substitutions (especially problematic when Ks = 0) and hence, a limited statistical power. To circumvent this major problem, some authors have replaced the Ks value by the substitution rate of neighboring introns, noted Ki [2]. Other authors have used a multi-species approach to increase the power of detecting PSG along specific branches of the hominid, primate or mammalian tree [11], [14]. Another interesting approach that does not require Ka/Ks>1 over the entire sequence but rather needs a fraction of codons to be positively selected was used by Nickel et al. (2008), based on the branch + site test of positive selection [15]. Also, Mann-Whitney U (MWU) tests were introduced to analyze GO terms enrichment in putatively PSG displaying small, yet not always significant *P*-values for positive selection [11]. Nevertheless, these various approaches are accompanied by quite conservative statistical significance tests with relatively weak power to detect positive selection [15].

Estimating Ka/Ks over full-length CDS to assess selective pressure contributes to the conservativeness of most methods, as positive selection may act only on a subset of codons. Multiple species sequence comparisons indicate that very few genes display Ka/Ks>1 for the entire CDS [10], [16]. Moreover, data from *Drosophila* indicate that positively selected codons tend to be clustered along the coding sequence [17], suggesting that the functional units along the protein might undergo different selective pressures. In several instances it has been shown that only specific protein domains or regions evolve under positive selection. For example, positive selection drives the evolution of only the extracellular domains in the lymphocyte CD45 protein of Old World monkeys (Filip and Mundy, 2004), as well as in the mammalian chemokine receptors of the CC type (Metzger and Thomas, 2010). Therefore, assessing selective pressure on the basis of protein domains or sub-regions instead of full-length CDS is a well-suited approach that may give more power to the tests.

To better understand how selection drives the evolution of coding sequences in the genome, it might be relevant to assess the amount of neutrally-evolving coding sequence. This is because adaptive evolution may depend on the amount of standing genetic variation on which selection may act, and genetic variation is substantially more abundant in neutrally-evolving regions. Alleles that are apparently neutral in an initial environment may confer a selective advantage in a new environment and therefore be positively selected. Such a situation has been nicely documented in sticklebacks in which alleles responsible for low-plated phenotypes are present at low frequency in marine population and have been consistently selected in most freshwater locations around the world [18].

Although major advances have been made in the understanding of how positive selection shapes the evolution of protein-coding genes at a genome-wide scale in mammalian representatives, very few studies have approached this issue on other vertebrate lineages. This was initially due to the lack of available genomes of related species but current technological advances in high throughput sequencing and improvements in *de novo* assembly methods [19] are providing new exomes for inter-specific comparisons. Accompanying the publication of the full genome of *Tetraodon*, an assessment of selective pressures acting on a reduced set of genes was performed for the first time in a non-mammalian vertebrate, yet without any focus on positive selection [20].

To test whether major patterns of positive selection discovered in mammals are also valid for teleost fishes, I present a genome-wide analysis of the selective pressure acting on the puffer fish lineage by comparing the protein-coding genes of *Tetraodon nigroviridis* and *Takifugu rubripes* (Teleostei, Tetraodontidae). I used an alternative approach to measure selection consisting in the calculation of the Ka/Ks ratio on sliding windows of fixed size for the entire set of orthologous genes of the two puffer fishes. This approach is motivated by the fact that positive selection most often acts on a subset of codons that tend to be clustered in a region along the sequence and therefore can be detected by a sliding window approach. This method, which has been used to detect positive selection in gene sub-regions [21], [22], has the advantage of requiring no more than two closely related species. Moreover, I present here the first genome-wide assessment of neutrally-evolving regions within protein-coding genes. Interestingly, the results of this analysis suggest that genes undergoing positive selection are a subset of genes displaying neutrally-evolving regions.

## Results

The 16,950 protein-coding orthologous genes that were alignable over more than 10% of their sequence between the *Takifugu* and the *Tetraodon* represent 86.5 % of *Tetraodon* protein-coding genes (assembly 4.0; Genebuild update May 2010) and 91.5% of *Takifugu* protein-coding genes (assembly 8.0; Genebuild update May 2010). The sum of all pairwise alignments resulted in 8,396,911 codons (not counting gaps). The alignment slicing procedure with sliding windows of 100 codons and a window shift of 50 codons generated 166,690 windows with less than 50% gap positions.

**Figure 1:**
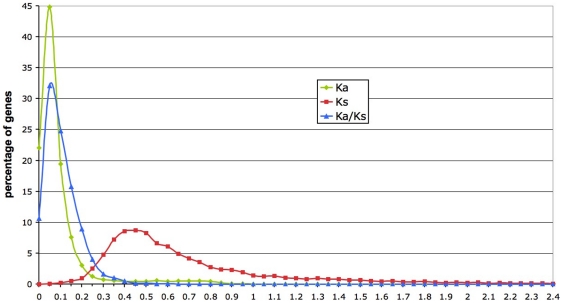
Distribution of Ka, Ks and Ka/Ks. These values are based on full-length CDS of *Tetraodon* and *Takifugu* orthologous genes. The gMYN method [55] was used. Genes were grouped into categories of 0.05 units. The X axis was cut at 2.4 for clarity purpose, yet the maximum Ka value reached 1.2, the maximum Ks category was 5 and the maximum Ka/Ks category was 7.

For the full-length CDS data set, the distribution of Ka values is very narrow, with 95% of genes displaying a Ka below 0.25. The median is 0.0548 where about 45% of genes are found (fig. 1). The distribution of Ks is more flat, with a median of 0.5875 and with a more pronounced right tail. The distribution of the Ka/Ks ratio shows that more than 95% of the genes display a ratio below 0.4; the median is 0.0875 and the mean is 0.107 (fig. 1).

### Genes displaying positive selection

The analysis of positive selection for full-length CDS revealed only three genes displaying a Ka/Ks ratio significantly higher than one (0.02% of all analyzed genes). Two of these genes encode immunoglobulin kappa constant proteins (ENSTNIP00000003454 and ENSTNIP00000001186), the third one is a novel protein with unknown function (ENSTNIP0000000055). Another three genes displayed a Ka/Ks ratio higher than one yet not significantly.

The analysis of the sliding windows dataset resulted in 287 genes with at least one window displaying Ka/Ks>1. However, only 33 windows showed a ratio significantly higher than one, totalizing 2,601 codon positions (0.031% of all analyzed codons). These windows were distributed over 31 different genes, which I called "partially Positively Selected Genes" (pPSG) (0.18% of all analyzed genes).

The 31 pPSG were scrutinized for chromosome location, functional annotation and GO terms (Table S1). Pfam domains were found in 23 genes, highlighting putative gene function. Furthermore, one or more GO terms were retrieved from Ensembl for 18 out of the 31 pPSG.

Chromosomal locations of pPSG in the *Tetraodon* genome indicate no particular clustering along chromosomes. However, the number of pPSG per chromosome was not correlated with the number of genes per chromosome (Spearman Rank correlation; *P* = 0.06), indicating that some chromosomes are enriched with pPSG. Chromosome 21 has the highest number of pPSG although it is small and gene-poor (according to the *Tetraodon* genome assembly 4.0 of Ensembl). This overrepresentation of pPSG can account for the lack of correlation between the number of pPSG and the number of genes per chromosome (when excluding chromosome 21 from the analysis, the correlation becomes significant). Because recently duplicated genes may preferentially undergo positive selection, I have checked if chromosome 21 was enriched with duplicated genes. In the analysis of teleost Whole-Genome Duplication (WGD) based on the *Tetraodon* genome [20], chromosome 21 was shown to carry notably fewer ancient duplications dating back to the WGD, relative to the number of genes per chromosome (highest estimated depletion of ≈2.4 times if we assume that the number duplicated genes per chromosome is proportional to the number of genes per chromosome). However, it has been shown that the *Tetraodon* has undergone subsequent series of gene duplications ranging from 4 to 70 Myr ago, that is, more recent than the WGD [23]. By using the set of 46 recently duplicated genes reported by Christoffels et al. (2006) and their chromosomal location, a ≈ 2.9 fold enrichment in recently duplicated genes can be measured for chromosome 21 (assuming a proportional number of duplicated genes relative to the total number of genes per chromosome). This result may support the proposal that the enrichment of chromosome 21 in pPSG is a consequence of an accumulation of recently duplicated genes that may be preferential targets for positive selection. A direct way to verify this suggestion is to calculate the Ka/Ks of the duplicated genes reported by Christoffels et al. [23], located on chromosome 21. Surprisingly, none of these genes displayed a Ka/Ks>1. This suggests either that the list of recently duplicated genes of Christoffels et al. [23] is not exhaustive and that other unidentified duplicated genes explain this pattern, or that the reason lies on other factors.

### GO analysis of positively selected genes

I examined if GO terms were enriched with pPSG by using the GOEAST software (Zheng and Wang, 2008). The analysis resulted in the identification of five significantly overrepresented Biological Process (BP) GO terms belonging to four independent GO lineages: "response to stimulus" (GO:0050896), "immune response" (GO:0006955), "mismatch repair" (GO:0006298) and "phosphate transport" (GO:0006817) (table 1; multi-test adjustment with *P*<0.05). Within the response to stimulus descendent terms, "defense response" (GO:0006952) was also significantly enriched with pPSG.

**Table 1:** Biological Process GO terms enriched with either pPSG, with genes displaying small *P*-values as given by the MWU test, or with GNR.

BP GO ID	Biological Process term	pPSG	MWU test	GNR
GO:0009889	Regulation of biosynthetic process			4.1e-2
GO:0032268	Regulation of cellular protein metabolic process			3.3e-8
GO:0006417	Regulation of translation			1.9e-7
GO:0006446	Regulation of translational initiation			6.2e-8
GO:0010468	Regulation of gene expression			4.9e-2
GO:0010608	Postranscriptional regulation of gene expression			1.8e-6
GO:0050896	Response to stimulus	2.4e-2	**1.26e-04**	
GO:0006950	Response to stress		1.2e-2	
GO:0006952	Defense response	2.4e-2		
GO:0002541	Activation of plasma proteins involved in acute inflammatory response			5e-3
GO:0002376	Immune system process		2.142e-3	
GO:0006955	Immune response	3.1e-2	2.147e-3	
GO:0002682	Regulation of immune system process			
GO:0002684	Positive regulation of immune system process			5e-3
GO:0050776	Regulation of immune response			5e-3
GO:0050778	Positive regulation of immune response			5e-3
GO:0002253	Activation of immune response			5e-3
GO:0019882	Antigen processing and presentation			5.1e-8
GO:0006956	Complement activation			5e-3
GO:0008152	Metabolic process			
GO:0006298	Mismatch repair	1.9e-2		
GO:0006302	Double-strand break repair			1.4e-2
GO:0016999	Antibiotic metabolic process			8.9e-3
GO:0022610	Biological adhesion			2.5e-10
GO:0007155	Cell adhesion			2.5e-10
GO:0007010	Cytoskeleton organization			4e-3
GO:0000226	Microtubule cytoskeleton organization			2.1e-3
GO:0006810	Transport			
GO:0015674	Di-, tri-valent inorganic cation transport			1.1e-2
GO:0006816	Calcium ion transport			1.2e-2
GO:0006820	Anion transport			3.3e-10
GO:0015698	Inorganic anion transport			6.5e-13
GO:0006817	Phosphate transport	3.5e-2		3.2e-21
GO:0007165	Signal transduction		2.272e-2	
GO:0006813	Potassium ion transport		4.9833e-2	
GO:0006355	Regulation of transcription, DNA-dependent		2.848e-2	
GO:0006468	Protein amino acid phosphorylation		2.8506e-2	

For the MWU test, *P*-values that are significant after Holm correction are given in bold.

Six Molecular Function (MF) GO terms showed an excess of pPSG (table 2). Two terms belong to the "protein binding" division: "mismatched DNA binding" (GO:0030983) and "interleukin-1 receptor binding" (GO:0005149). Two other terms are part of the "catalytic activity" division: "adenylate cyclase activity" (GO:0004016) and "cis-trans isomerase activity" (GO:0016859). The last enriched term is high in the hierarchy: "extracellular matrix structural constituent" (GO:0005201).

**Table 2:** Molecular Function GO terms enriched with either pPSG, with genes displaying small *P*-values as given by the MWU test, or with GNR.

MF GO ID	Molecular Function term	pPSG	MWU test	GNR
GO:0005515	Protein binding			2.3e-2
GO:0030983	Mismatched DNA binding	4.7e-3		
GO:0005149	Interleukin-1 receptor binding	3.4e-3		
GO:0003712	Transcription cofactor activity			2.6e-2
GO:0017016	Ras GTPase binding			9.6e-3
GO:0005249	Voltage-gated potassium channel activity		4.97e-2	
GO:0043167	Ion binding		4.93e-2	
GO:0043169	Cation binding		4.9e-2	
GO:0046872	Metal ion binding		4.9e-2	
GO:0046914	Transition metal ion binding			8.8e-3
GO:0008270	Zinc ion binding			6.2e-5
GO:0030247	Polysaccharide binding			3.4e-3
GO:0005540	Hyaluronic acid binding			1.7e-3
GO:0003676	Nucleic acid binding			1.1e-5
GO:0003743	Translation initiation factor activity			8.6e-5
GO:0008227	G-protein coupled amine receptor activity			1.3e-3
GO:0004969	Histamine receptor activity			1.9e-2
GO:0003824	Catalytic activity			
GO:0004016	Adenylate cyclase activity	9.7e-3		
GO:0016859	Cis-trans isomerase activity	1.1e-2		
GO:0004869	Cytein-type endopeptidase inhibitor activity			5.9e-4
GO:0005199	Structural constituent of cell wall	1.1e-2		2.4e-11
GO:0005201	Extracellular matrix structural constituent	4.6e-3		5.8e-6

Finally, six Cellular Component (CC) GO terms were significantly enriched with pPSG, all belonging to the "extracellular region" (GO:0005576) division and notably the "extracellular matrix" (GO:0031012) (table 3).

**Table 3:** Cellular Component GO terms enriched with either pPSG, with genes displaying small *P*-values as given by the MWU test, or with GNR.

CC GO ID	Cellular Component term	pPSG	MWU test	GNR
GO:0005576	Extracellular region	1.76e-2		
GO:0044421	Extracellular region part	9.9e-3	3.04e-2	4.39e-6
GO:0031012	Extracellular matrix	9.8e-3		1.8e-6
GO:0005578	Proteinaceous extracellular matrix	9.9e-3		8.23e-5
GO:0044420	Extracellular matrix part	9.9e-3		2.74e-9
GO:0005581	Collagen	9.88e-3		8.86e-12
GO:0016020	Membrane			
GO:0042611	MHC protein complex			6.59e-6
GO:0042612	MHC class I protein complex			1.45e-3
GO:0042613	MHC class II protein complex			4.14e-3
GO:0034704	Calcium channel complex			7.87e-3
GO:0005891	Voltage-gated calcium channel complex			7.87e-3
G0:0005622	Intracellular			1.77e-2
GO:0030134	ER to Golgi transport vesicle			1.07e-2
GO:0012507	ER to Golgi transport vesicle membrane			1.07e-2
GO:0030127	COPII vesicle coat			1.07e-2
GO:0042579	Microbody			4.09e-2
GO:0005777	Peroxisome			4.09e-2

Among the 256 genes displaying windows with Ka/Ks>1, albeit not always with a significant *P*-value, many of them showed small *P*-values close to the significance level. The Mann-Whitney U test was used to assess whether some GO terms were enriched with genes displaying small *P*-values, which may be undetected pPSG (due to a conservative statistical test or to a non-optimal window positioning). Within the BP category, eight enriched terms were found, notably: "response to stimulus" (GO:0050896) and "response to stress" (GO:0006950), "immune system process" (GO:0002376) and "immune response" (GO:0006955), "signal transduction" (GO:0007165), and "regulation of transcription DNA-dependent" (GO:0006355) (table 1). Four enriched GO terms were found within the MF category: "voltage-gated potassium channel activity" (GO:0005249), "ion binding" (GO:0043167) and two of its descendent terms, "cation binding" (GO:0043169) and "metal ion binding" (GO:0046872) (table 2). As to the CC category, the single enriched GO term was "extracellular region part" (GO:0044421) (table 3).

A striking result is the significant excess of pPSG outside the cell, as revealed by the enriched MF GO term "extracellular matrix structural constituent" (table 2) and the CC term "extracellular region" and some of its descendent terms (table 3). I checked whether this finding was specific to the puffer fishes or if it was a more general pattern for vertebrates. I used the recently published set of 400 genes that underwent positive selection along the phylogeny of six mammalian representatives [11], and checked for GO terms enrichment against human and mouse GO gene-association databases, as implemented in GOstat (see materials and methods). The analysis of GO terms belonging to the CC category indicated that the terms "extracellular region part" (GO:0044421) and "extracellular space" (GO:0005615) were significantly enriched with PSG (table 4). In addition, several GO terms related to cell surface, membrane and plasma membrane showed a significant excess of PSG. The few intracellular GO terms enriched with PSG belong to the "vacuole" hierarchy (GO:0005773) and the gene products form part of the "lysosome" (GO:0005764). Lysosomes are organelles where hydrolytic enzymes degrade waste and foreign material such as bacteria, at a low pH. The lysosomal membrane protects the rest of the cell from the degradative enzymes located inside this very specialized cellular compartment; the lysosomal membrane also plays a role in plasma membrane damage repair by serving as a membrane patch. These characteristics link the lysosome to the plasma membrane and also to cell defense response and therefore may explain the overrepresentation of PSG.

**Table 4:** Cellular Component GO terms enriched with PSG in human and mouse.

GO term ID	Cellular Component GO term	Human	Mouse
GO:0044421	Extracellular region part	1.69E-07	6.27E-08
GO:0005615	Extracellular space	2.62E-05	6.27E-08
GO:0044464	Cell part	2.16E-05	_
GO:0009986	Cell surface	2.22E-06	1.97E-11
GO:0009897	External side of plasma membrane	1.49E-08	1.97E-11
GO:0016020	Membrane	1.58E-28	4.04E-07
GO:0044425	Membrane part	1.35E-35	1.52E-07
GO:0031224	Intrinsic to membrane	9.10E-41	6.27E-08
GO:0016021	Integral to membrane	7.28E-41	6.27E-08
GO:0005886	Plasma membrane	9.10E-41	3.28E-06
GO:0044459	Plasma membrane part	3.32E-20	7.50E-06
GO:0031226	Intrinsic to plasma membrane	2.38E-26	_
GO:0005887	Integral to plasma membrane	1.62E-25	_
GO:0043235	Receptor complex	1.19E-05	1.37E-04
GO:0042101	T cell receptor complex	0.0014	6.83E-04
GO:0042105	Alpha-beta T cell receptor complex	_	1.82E-04
GO:0008305	Integrin complex	_	0.0352
GO:0001772	Immunological synapse	5.65E-06	2.09E-05
GO:0016324	Apical plasma membrane	0.0266	_
GO:0005773	Vacuole	1.17E-04	3.84E-04
GO:0000323	Lytic vacuole	3.17E-05	1.56E-04
GO:0005764	Lysosome	3.17E-05	1.56E-04
GO:0005765	Lysosomal membrane	_	0.015

The set of PSG were taken from [11] and analyzed against the human and mouse gene-associated GO database as implemented in GOstat [58].

### Genes with neutrally-evolving regions

A total of 4,511 windows displayed a Ka/Ks ratio not significantly different from one (neutrally or nearly neutrally evolving regions), totalizing 317,011 codon positions (3.8% of all analyzed codons). These windows were distributed over 2,530 different genes (14.9% of all analyzed genes), which I refer to as Genes with Neutrally-evolving Regions (GNR). About 40% of GNR showed two or more neutrally-evolving windows and up to 19 windows.

GNR were found to be significantly overrepresented in 25 GO terms within the BP category, belonging to nine different hierarchies (table 1). The most significant are: "regulation of cellular protein metabolic process" (GO:0032268), "postrancriptional regulation of gene expression" (GO:0010608), "antigen processing and presentation" (GO:0019882) involved in the immune response, "cell adhesion" (GO:0007155), "organelle assembly" (GO:0070925) and "phosphate transport" (GO:0006817).

In the MF category, fourteen GO terms were enriched with GNR (table 2), belonging to the following eight lineages: "protein binding" (GO:0005515), "transition metal ion binding" (GO:0046914), "polysaccharide binding" (GO:0030247), "nucleic acid binding" (GO:0003676), "G-protein coupled amine receptor activity" (GO:0008227), "cystein-type endopeptidase inhibitor activity" (GO:0004869), "structural constituent of cell wall" (GO:0005199), "extracellular matrix structural constituent" (GO:0005201). The two last terms being enriched the most.

Among the CC terms, sixteen displayed an excess of GNR (table 3). They are organized into five different hierarchies: "extracellular region part "(GO:0044421), which displayed the highest overall enrichment, "MHC protein complex" (GO:0042611) located in the cell membrane, "calcium channel complex" (GO:0034704), "ER to Golgi transport vesicle" (GO:0030134), and "microbody" (GO:0042579).

As neutrally-evolving DNA sequences can accumulate more mutations, they may be essaying a wider range of functional alternatives, increasing the opportunity of finding an advantageous allele that will undergo positive selection. I have tested this hypothesis by comparing the list of pPSG and of GNR and I found that the majority (77.4%) of pPSG are also GNR, i.e. they also have one or more windows evolving neutrally. This is 5.4 fold higher than expected by chance, indicating a strong link between positively selected and neutrally-evolving gene regions.

## Discussion

### Strength of selection

In the comparison between *Takifugu* and *Tetraodon*, the strength of selection acting on protein-coding genes is given by an overall Ka/Ks ratio of 0.107, based on full-length CDS. This result is slightly lower than the ratio of 0.14 previously reported for these fishes [20]. The difference can be explained in part by the dataset used: our analysis is based on 16,950 orthologous genes pairs while Jaillon et al. (2004) used about 13,000 genes. However, the methods used for estimating Ka/Ks may account for most of the difference. Indeed, Jaillon et al. (2004) used the PBL method [24], [25] that does not correct for the bias in base frequency. Methods that do not fully account for this bias overestimate Ka/Ks when this ratio is lower than one [26], which may explain the higher value reported by Jaillon et al. (2004).

As compared to mammalian species, the overall Ka/Ks ratio of the puffer fishes is about the same as the one estimated for the murid lineage (mouse and rat; Ka/Ks ≈ 0.14), and markedly lower than the one reported for the human-chimpanzee lineage (Ka/Ks ≈ 0.23) [2], [16]. These comparisons indicate that there is a 50–60% excess of amino-acid-changing mutations in the human-chimpanzee lineage relative to the murid and puffer fish lineages, respectively. The accelerated evolution of proteins in hominids has been explained by the nearly neutral theory of evolution [27] that predicts that selection against deleterious mutations is less efficient when effective population size (*N*) is small. The presumed larger population size of puffer fishes and murids may thus explain their lower rate of protein evolution as compared to human and chimpanzee.

The number of PSG may depend on the age of divergence of the analyzed species. In a comparison between human and chimpanzee, two species that diverged 4-5 My ago [28], [29], Nielsen et al. (2005) reported 35 genes with a significant signal of positive selection out of 8,079 analyzed genes (0.43%). However, Kosiol et al. [11] found a smaller proportion by using the more accurate branch model for detecting positive selection [30]. They detected 10 and 18 PSG in the human and chimpanzee branches, respectively, leading to 28 PSG in the hominid lineage out of 14,558 analyzed genes (0.19%). This same study reported a higher proportion of PSG (0.56%) in the clade of mouse and rat, two species that diverged 16–23 My ago [31] (61 PSG out of 10,763 analyzed genes). In the comparison of *Tetraodon* and *Takifugu*, which split ∼18–30 My ago [32], 31 pPSG were detected over a total of 16,950 analyzed genes, resulting in a similarly small proportion of PSG (0.18%) as compared to the hominid lineage, but smaller than that in the murid clade. It is not clear whether the latter difference is significant given that the age of divergence of mouse-rat and *Tetraodon*-*Takifugu* is comparable. However, it is important to note that the different methods used for inferring positive selection and their associated significance tests might explain part of this difference.

The proportion of pPSG may vary across chromosomes and our results suggest that *Tetraodon* chromosome 21 is enriched with pPSG. According to our current understanding, such an enrichment may be due either to (i) the presence of a cluster of genes involved in a biological function targeted by positive selection (like immune response for instance), (ii) an enrichment in genes that do not play central biological functions, or (iii) an overrepresentation of recently duplicated genes that initially had redundant information and subsequently, for some of them, were neofunctionalyzed via adaptive evolution. This last statement meets the previously proposed concept that a network of genes of recent origin may explain an excess of positive selection in a given biological process [13]. For the moment, the data at hand do not support neither an enrichment with genes belonging to a same biological function nor an overrepresentation of recently duplicated genes, while the scarce annotations of our pPSG do not allow an assessment of the importance of the biological functions of the pPSG (Table 1). Further investigations are needed to clarify this issue.

### Mammalian *versus* fish GO analyses of pPSG

With the growing amount of gene annotations in whole genome databases, and in particular in GO information, it was possible to perform a customized search for GO term enrichment with pPSG on *Tetraodon*. Several GO terms were enriched with pPSG and many of them were retrieved with the MWU test of excess of small *P*-values associated to the test for positive selection. As more genes were included in the MWU test, additional GO terms were highlighted with this method. To uncover the patterns of positive selection that are shared between mammals and the teleost fish lineage analyzed here, I have compared the set of enriched GO terms that I obtained with either of the two methods (i.e. GO term enrichment based on pPSG or based on MWU tests of *P*-values) and the set of enriched GO terms reported for mammals (Kosiol et al. 2008) and for humans (Nickel et al. 2008) (table 5). More than half of the BP terms are shared between mammals and puffer fishes, including notably the immune and defense response, signal transduction and regulation of transcription. BP and MF terms that show an excess of PSG in mammals but not in fishes include "sensory perception", "neurological process", "cytolysis", "single fertilization" and some catalytic activities like transferase and serine hydrolase. Notable puffer fish specific terms include "mismatch repair" and "extracellular matrix structural constituent" (table 5).

**Table 5:** Summary of main GO terms enriched in PSG that are shared between mammals and puffer fishes, that are specific to mammals or specific to puffer fishes.

Main GO terms	Shared by mammals and fishes	Missing in fishes	Specific to fishes
**Biological process**	X		
Response to stimulus	X		
Defense response	X		
Response to stress* or to wounding**	X		
Immune system process, Immune response	X		
Signal transduction	X		
Regulation of transcription DNA-dependent	X		
Ion transport ***	X		
Sensory perception		X	
Neurological process		X	
Cytolysis		X	
Single fertilisation		X	
Mismatch repair			X
Protein phosphorylation			X
**Molecular fonction**			
DNA binding** or Mismatch DNA binding*	X		
Protein binding**, Chemokine receptor binding**, Interleukin binding**, Interleukin-1 receptor binding *	X		
Metal ion binding	X		
Olfactory receptor activity, Rhodopsin-like receptor activity, Taste receptor activity		X	
Protease inhibitor activity		X	
Transferase activity, RNA methyltransferase activity		X	
Nucelotide binding		X	
Serine hydrolase activity		X	
Voltage-gated potassium channel activity			X
Adenylate cyclase activity			X
Cis-trans isomeras activity			X
Structural constituent of cell wall			X
Extracellular matrix structural constituent			X
**Cellular component**			
Extracellular region, Extracellular matrix*, Extracellular space**	X		
Membrane, Intrinsic to membrane, MHC protein complex		X	
Nucleus		X	
Cytoplasm		X	

*Found in puffer fishes.

**found in mammals.

***the type of ion may differ between mammals and puffer fishes.

The data for mammals has been taken from [11] and from [10]. Some GO terms were grouped if they were close descendent/parent terms in the GO database hierarchy.

The difference between mammals and fishes in sensory perception via olfaction, taste, and view is of particular interest. Mammals have developed highly specialized ways of sensing their environment with various lineage specific refinements. Rodents have largely enriched their pheromone and odorant receptor repertoires [33], [34], [35] while primates have undergone strong positive selection on genes related to taste, color vision and also olfaction (Mikkelsen et al., 2005; Kosiol et al., 2008). On the other hand, fishes have to detect and discriminate among an enormous array of compounds present in their aquatic environment, a large fraction of them being relatively unspecialized metabolic products [36]. It has been suggested that in such a complex background of organic and inorganic compounds, there is little pressure to evolve specialized chemicals and their receptors to communicate among organisms but rather chemoreception requires the discrimination of small differences in the mixture composition [37]. Therefore, although the senses of smell and taste are quite developed in fishes, they are not particularly targeted by positive selection. As to color vision, most teleost fishes have four spectrally distinct classes of cone opsins while only two classes are present in most eutherian mammals, supporting a dichromatic color vision in the latter [38]. In marine mammals and in some nocturnal species, an additional class of cone opsin has been lost leading to a monochromatic vision. But the most notable departure from the mammalian dichromatic color vision is the re-evolution of trichromacy in primates either *via* gene duplication, as in Old World primates (including human), or through gene polymorphism as found in New World monkeys (reviewed in [39]). The independent and recent re-evolution of trichromacy in these two main groups of primates most likely explains why receptor activity related to color vision has been detected to be enriched with PSG in a mammalian scan with a strong contribution of primates [11]. However, recent studies of colorful fishes have revealed positive selection acting on lineage specific duplicated genes encoding visual pigments [40], [41] and therefore, our result based on puffer fishes may not hold true for all teleost fishes.

My comparative analyses indicated that the gene set encoding extracellular proteins is enriched with PSG in puffer fishes as well as in mammals (table 5). This finding suggests that the most general location where adaptive evolution occurs is outside the cell rather than inside the cell. This result coincides with the observation that mammalian genes encoding proteins that are exported evolve faster than genes whose products reside inside the cell, and that this intra- versus extracellular location has a larger effect on protein sequence evolution than any other factor proposed previously [6]. Moreover, the same authors found that genes encoding extracellular proteins, and to a lesser extent genes encoding plasma membrane proteins with an extracellular domain, have on average a higher Ka/Ks ratio than genes encoding intracellular proteins, suggesting stronger purifying selection acting on genes encoding intracellular proteins. I have tested whether this trend is also recovered in the *Takifugu*-*Tetraodon* lineage. Mean Ka/Ks ratio is significantly higher in genes encoding extracellular proteins (GO:0005576) than in genes encoding intracellular proteins (GO:0005622) (permutation test of mean equality; 10,000 permutations; *P* = 9.9e-5). The Ka/Ks ratio was on average also higher for genes encoding cell surface proteins (GO:0009986) than genes encoding intracellular proteins, yet not significantly. Interestingly, these results indicate that in mammals as well as in fishes, genes encoding proteins targeted to the extracellular environment undergo more often positive selection and their proteins have an accelerated evolutionary rate than genes encoding intracellular proteins. Genes encoding plasma membrane proteins seem to have an intermediate status between those encoding proteins targeted to extra- or to intracellular compartments.

I speculate that the conserved nature of genes encoding intracellular proteins can be partly explained by the stable intracellular environment in which functional optima have already been reached with time and new variations, that have good chances to perturb the complex and rich network of intracellular protein interactions, are generally discarded by strong purifying selection. On the other hand, many adaptive functions take place at the periphery or outside the cell, like for instance processes involved in the immune and defense response mediated in particular by apoptosis. Apoptosis related genes have been shown to be enriched with PSG that are essentially located upstream on the cascade of events, at the level of circulating signal molecules and their plasma membrane receptors [13]. The correspondence found in primates and other mammals between the excess of PSG in immune and defense responses and the excess of PSG encoding extracellular proteins is consistent with what I found in the *Tetraodon-Takifugu* lineage. Nevertheless, our results indicate that in fishes, collagens, that are structural constituents of the extracellular matrix, are also contributing to the enrichment of PSG in the extracellular region. This fish specific trait may be explained by the more diverse and numerous collagens present in fishes as compared to mammals [20]. To verify the generality of this proposal, I exported from Ensembl all the members of the collagen gene family (Pfam PF01391) of several mammals (human, macaque, mouse, rat, caw, dog, panda, horse) and fishes (zebrafish, medaka, stickleback, *Takifugu* and *Tetraodon*). The comparison of these two sets of collagen gene lists revealed that the mean number of collagens in fishes (mean = 100.4) is significantly higher than in mammals (mean = 80) (*T*-test; *P* = 0.0063), giving more support to this explanation.

### Relationship between neutral evolution and positive selection

Are neutrally-evolving gene regions more prone to undergo positive selection than gene regions evolving under purifying selection? The reasoning underlying this question is that neutrally-evolving gene regions experience more non-synonymous mutations than negatively selected regions and might therefore end up more often by mutating into an advantageous allele. The results presented here indicate that pPSG are more often than random also displaying neutrally-evolving parts. This suggests that a substantial proportion of positively selected regions emerged from an ancestral neutrally-evolving region. This hypothesis is also supported by the fact that GO terms enriched with pPSG are more often than random also enriched with GNR (Z-test; *P*<0.001). When comparing BP, MF and CC GO terms, this correlation is significantly more pronounced for the CC category, where most (83%) of the terms enriched with pPSG are also enriched with GNR (Test of homogeneity; chi-square = 6.54; df = 2; *P* = 0.038). This finding is in agreement with the fact that genes encoding proteins targeted towards the extracellular region, which is the main GO term enriched with pPSG of the CC category, are significantly less constrained in their evolution and are more prone to undergo positive selection.

The relationship between neutral standing variation and positive selection has been considered theoretically [42], [43] but its role in the evolution of natural populations is still poorly documented. Perhaps the best example is given by the threespine sticklebacks that recently moved from marine environments and colonized freshwater habitats in several locations. These new populations underwent a parallel reduction of their body armor due to the adaptive-driven increase in frequency of the responsible allele, which is found at low frequency in marine populations [18]. Adaptive evolution emerging from standing neutral mutations has also been demonstrated by directed evolution, an engineering strategy for exploring and improving protein function. In this way, it has been shown that neutral mutations that increase protein stability can lead to adaptive evolution by allowing a subsequent beneficial but destabilizing mutation [44].

Also, by altering a non-central activity of a protein (also called "promiscuous" function), a neutral mutation can provide a starting point for subsequent beneficial mutations [45], [46].

It is also possible to provide here an assessment of how much neutrally-evolving positions are needed for giving rise to positively selected variants. The estimates on the number of codons involved in positively selected windows (2,601 codons) and in neutrally-evolving windows (317,011 codons) suggest that out of every 120 neutrally-evolving codons, one will undergo positive selection. This very small proportion of adaptive changes is in agreement with the most recent estimate calculated for human and chimpanzee. While Zhang and Li [47] found no evidence of adaptive amino acid substitutions, other studies reported estimates varying between 0 and 8%, depending on the method and the dataset used [2], [48].

The results presented here are based on a sliding window analysis of Ka/Ks between homologous gene pairs. This approach has the advantage of needing only a pair of species and can reveal positive selection acting on gene sub-regions. I have used here a window size of 300 nucleotides ( = 100 amino acids) and a window shift of 150 nucleotides to perform a relatively fine scan of gene sub-regions. Changing the window size or shift mode will not question the pPSG determined here but may allow the detection of additional pPSG. For a complete detection of pPSG, future works will certainly be oriented toward analyzing relevant windows of variable size.

Further investigations based on the growing number of full genome, transcriptome and exome projects will rapidly increase our understanding of patterns of positive selection and adaptive evolution in the entire vertebrate lineage and beyond. It is also of major interest to investigated the patterns of neutrally-evolving gene regions to better characterize the relationship between neutral evolution and the emergence of pPSG. If neutrally-evolving gene regions can lead more frequently to positively selected alleles, the ensuing prediction is that genomes bearing more GNR will display more pPSG. This prediction will be tested by future analyses of GNR and PSG in more genomes.

## Materials and Methods

Orthologous genes of *Tetraodon* (*Tetraodon nigroviridis*; assembly TETRAODON 8.0; Genebuild update May 2010) and *Takifugu* (*Takifugu rubripes*; assembly FUGU 4.0) were downloaded from Ensembl (http://www.ensembl.org) using the BioMart tool and the Ensembl Genes 58 dataset. New evidence of orthology has been recently given by the first integrated genome map of *Takifugu* which reveals a strong conservation of synteny between *Takifugu* and *Tetraodon* [49]. The initial number of orthologous genes was 22,426. Some *Tetraodon* genes had no *Takifugu* counterpart and vice versa; they were thus discarded. Several genes were present more than once due to the presence of recently duplicated genes in one or the other species. In those cases I kept the gene pair with the highest sequence similarity, as given in the BioMart output. Gene pairs alignable only partially resulted in a low percentage of sequence identity over their entire sequence. To ensure that the analyzed gene pairs are not only comparable over a small fraction of their sequence and hence may be of doubtful homology, I considered further only pairs with more than 10% sequence (as given in the BioMart output). Using this cleaned list of gene pairs, I downloaded from Ensembl Genes 58 dataset the corresponding CDS of the canonical transcripts (defined as either the longest CDS, if the gene has translated transcripts, or the longest cDNA [50]). Here, 25 additional genes showed stop codons in their CDS and were discarded from the analyses because they may represent erroneously predicted genes or exons. The final list comprised 16,950 orthologous gene pairs.

### Alignments

Using PyCogent scripts [51], the following pipeline was designed for preparing high quality alignments for downstream applications. First, the CDS of the canonical transcripts were imported from Ensembl. Accurate pairwise alignments were obtained by translating the CDS and aligning the amino acid sequences using the Needleman-Wunsch method [52]. Then amino acid alignments were back translated onto their corresponding nucleotide alignments.

The selective pressure acting on sub-regions of the coding sequence of each gene was assessed using a sliding windows approach, a method that slices the pairwise alignment into windows of uniform, user-defined size. Windows are shifted along the pairwise alignment according to a user-defined shift length. Here, I analyzed windows of 300 nucleotides long ( = 100 codons) and with a window shift of 150 nucleotides ( = 50 codons). The slicing of the CDS into sliding windows was performed using the program Split of the KaKs_calculator 2.0 package [53], resulting in a file containing the pairwise alignments of all the windows of all the orthologous genes. Before proceeding to the slicing, the pairwise alignments were elongated using gaps in order to reach a length divisible by 300. This procedure enables the inclusion of the last partition of alignments into the downstream analysis pipeline. The windows displaying less than 50% comparable positions, i.e. not composed by gaps in one or the other sequence, were discarded. This is because alignment regions containing numerous gaps were essentially due to a shorter CDS or a large indel in one of the two species. In such situations, the Needleman-Wunsch pairwise alignment method tends to accommodate the bordering codons into positions spread over the part which is absent in one of the sequences, resulting in non-homologous codon alignment with high similarity.

### Analyses of selective pressure

The selective regime under which each window of the protein-coding sequences has been evolving was assessed by using the MYN method [54], a modification of the Yang and Nielsen method [26]. I used the more realistic version of this method that incorporates heterogeneous rates across sites using a gamma distribution (gMYN), as developed by Wang et al. [55] and implemented in KaKs_calculator 2.0 [53]. The outputs were ordered by Ka/Ks values and by the significance level of the associated Fisher test, which indicates whether the Ka/Ks ratio is significantly different from one (Ka/Ks = 1 indicates neutral evolution). The genes displaying positively selected windows and those with neutrally-evolving windows were analyzed separately. The following information was recovered for genes with positively selected windows: chromosomal location in the *Tetraodon* genome, functional annotation and Gene Ontology (GO) associated terms as given in Ensembl (http://www.ensembl.org) and also Pfam domains (http://pfam.sanger.ac.uk/).

### GO analysis

Genes with GO annotations were retrieved from the Ensembl *Tetraodon* assembly 8.0 database, resulting in a set of 13,000 annotated genes which was used in further analyses as the customized *Tetraodon* GO annotation dataset. To check whether particular GO terms were overrepresented in the set of genes displaying positively selected regions or neutrally-evolving regions, I used the GOEAST software [56]. GOEAST uses a hypergeometric test to identify significantly enriched GO terms. To circumvent the multi-test problem, the raw *P*-values are by default adjusted using the Benjamini-Yekutieli method [57]. The hypergeometric test and the log-odds ratio were calculated by using the GO terms associated to genes in the query list and in the customized *Tetraodon* GO annotation dataset [56]. Searches were performed on the Biological Process (BP) category of the GO database (http://www.geneontology.org/) as well as on the Molecular Function (MF) and on the Cellular Component (CC) categories.

To compare our results with the patterns of GO term enrichment found in mammalian PSG [11], I performed GO term enrichment analyses using the set of mammalian PSG and analyzing them against the GO annotation dataset background of human and mouse using the GOstat software [58]. This software was chosen because it includes the GO gene-association databases of various mammals. The Benjamini and Hichberg [59] correction for false discovery rate was used.

In addition to analyses performed on genes with Ka/Ks significantly greater than one, I also tested whether individual GO terms had an excess of genes with small *P*-values for Ka/Ks>1. I used the (one-sided) Mann-Whitney U (MWU) test in which the distribution of *P*-values of genes belonging to a given GO term is compared to the one of genes not belonging to that GO term. In order to account for the tree-like structure of the GO database, the parent GO terms of the ones assigned to each gene were also considered. GO terms associated to at least three genes were included in this analysis. The *P*-values of the MWU test were also corrected for multiple comparisons using the Holm correction.

## Supporting Information

Table S1 **Chromosome location and functional annotation of the 31 Tetraodon genes harboring at least one window with significant positive selection (Ka/Ks ratio significantly higher than one).**
(XLS)
